# “On the books” yet “off the record”-occupational injury and migrant women: scoping review findings from OECD countries, with implications for New Zealand

**DOI:** 10.3389/fgwh.2024.1346834

**Published:** 2024-05-09

**Authors:** Kelly Radka, Emma H. Wyeth, Brooke Craik, Christina R. Ergler, Sarah Derrett

**Affiliations:** ^1^ Ngāi Tahu Māori Health Research Unit, Division of Health Sciences, University of Otago, Dunedin, New Zealand; ^2^ School of Geography, Division of Humanities, University of Otago, Dunedin, New Zealand

**Keywords:** migrant women workers, foreign-born women workers, occupational injuries, work-related injuries, scoping review, New Zealand

## Abstract

**Introduction:**

Little appears to be known regarding the work-related injury (WRI) experiences of migrants (those born in a country other than their identified host country) and specifically, women migrants.

**Methods:**

As part of a wider PhD project investigating the WRI experiences of New Zealand (NZ) migrants, a review of NZ mainstream media coverage of migrants WRIs was undertaken, which identified no representations of migrant women's WRI experiences. In turn, a scoping review was undertaken to identify peer-reviewed publications reporting empirical findings about WRI experiences and outcomes for migrants in Organization for Economic Co-operation and Development (OECD) member countries, including NZ. This paper aims to identify and describe findings for migrant women specifically. From 2,243 potential publications, 383 proceeded to full text review; ultimately 67 were retained. These 67 publications were reviewed to identify findings specifically for occupationally injured migrant women; 22 such publications (from 21 studies) were found. This paper reports: the characteristics of identified studies; characteristics of migrant women within; frameworks and theories used, and knowledge (and gaps) related to occupationally injured migrant women.

**Results:**

Publications came from only four OECD countries, the United States, Canada, Australia, and Spain. A range of study designs, and topic areas (working conditions, legal rights, identities, the role of gatekeepers, and precarity), were identified; however, only three studies reported findings for longer-term experiences and outcomes of WRIs. Nine publications considered theoretical models underpinning research, including theories about precarious work, stigmatization, and citizenship. However, there was a paucity of analyses of the WRI experience throughout the life-course, highlighting a gap in understanding of how these experiences are “lived” over the long term by occupationally injured migrant women.

**Discussion:**

Scoping review findings were synthesized using a provisional “matryoshka framing narrative” model, to be refined through forthcoming qualitative interviews with occupationally injured NZ migrant women. This model highlights the multitude of influences in WRI experiences, potentially specific to migrant women, suggesting the consequences of WRIs may be uneven, with migrant women experiencing different, and potentially, greater disparities in outcomes. These findings provide an impetus to investigate knowledge gaps and urgently address potential disparities in WRI outcomes for migrant women specifically.

## 1 Introduction

In New Zealand (NZ), 28.7% of the overall population were reported as non-NZ born (1) in 2020; an increase of 33% since 2011 (2). Alongside this, there is a high rate (83.5%) of workforce participation among foreign-born workers (3). Internationally, studies have highlighted a “funneling” of migrant workers into certain industries and occupation types (4), including “3-D jobs” characterized as: dirty, demanding, and dangerous (5) and potentially, discriminatory (6). Despite increasing numbers of migrants, high workforce participation, and participation in “risky” occupations, little appears to be known about the experiences and outcomes of work-related injuries (WRIs) of this group in NZ. However, some evidence suggests WRI inequities may exist. Firstly, the Accident Compensation Corporation (ACC) (NZ's no-fault personal injury insurance scheme) (7) reports WRI claims are disproportionately high amongst those identifying as “other ethnicity”, including Middle Eastern, Latin American, African, and other ethnicities (8). Importantly, this data is by ethnicity, and does not report outcomes specifically for migrants (i.e., among non-NZ-born workers).

Further, the Prospective Outcomes of Injury Study (POIS) (9) reported less favorable injury outcomes for those identifying as “overseas born” at three months post-injury in NZ (10). A follow-up study identified associations between inadequate pre-injury household income and perceiving the injury as a threat to life, with long-term disability following injury for NZ migrants (11). These results, however, were for *all* injury types, not WRIs specifically. Additional research is needed to elucidate migrants' WRI experiences and outcomes, along with those of specific groups of migrants in NZ, including women.

Findings reported in this paper come from a larger PhD project (by the first author) investigating the experiences and outcomes of occupationally injured migrant workers in NZ comprising three components: a NZ media review, a scoping review of empirical publications from OECD countries (the focus of this paper) and follow-up qualitative interviews with occupationally injured migrant women. The review of mainstream media articles (12) was undertaken to understand media representations and broader societal views of migrants' WRIs in NZ. Through this, no mainstream media coverage of WRIs was identified for NZ migrant *women*, despite using a wide range of media sources and search terms, indicating the experiences of women may be particularly “hidden”. (12) Overseas media reviews have found WRIs being represented as occurring mainly amongst men and in primary industries (13, 14). Such representations potentially lead to tacit media framing of WRIs (by default) as an issue for male workers (13, 14). This is concerning as media is a recognized source of health information for the public (15, 16) with potential relationships between media and policy (17). A potential “default” conceptualization of WRIs in media could potentially contribute to unwarranted inattention on the WRI experiences of other groups, including women, and as identified in the NZ context, specifically on migrant women (12).

Attention on migrant women's WRIs is warranted as they may face specific challenges related to employment. For example, occupations such as caregiving and cleaning are characterized by precarious working conditions, frequently carried out away from the public eye (18, 19). Women may be assigned to duties perceived as “lighter” than those of male workers, however, these can be repetitious and may require greater dexterity, alongside being “on your feet” for long intervals (20). Injuries resulting from these duties may be: “…less likely to be recognized, validated, and compensated than work-related injuries arising from activities more frequently performed by men” (20).

Further, the WRI experiences of migrant women workers may be shaped by family responsibilities as primary caregivers of children (21), family separation (20, 22), and provision of remittances to support family remaining in the country of origin (23). These may occur alongside other recognized mechanisms of WRI disparity including gender (24–28), race (29–31), ethnicity (28, 31–34), immigration status (26, 35–37), socio-economic status (38, 39), educational background (40), and language proficiency (41, 42).

Following the NZ media review (12), including an identified paucity of *media* attention on the WRI experiences of NZ migrant women, a scoping review was undertaken, to investigate the extent and nature of *empirical* publications reporting WRI experiences and outcomes of migrants, internationally and in NZ. This paper focuses on studies reporting findings specifically for migrant *women,* identifying key areas for further research, some of which will be addressed in follow-up qualitative interviews with occupationally injured NZ migrant women as part of the larger project.

Prior to this scoping review, searches were undertaken in the Open Science Framework and BMJ Open journal (both known to register and publish scoping review protocols), and the SCOPUS database, to identify existing scoping reviews. Scoping reviews investigating migrants' WRI outcomes and experiences in Spain (43, 44) and Italy (43) were identified; none were identified as having either an “international”, or NZ context specifically (searches last run 25 August 2022).

## 2 Methods

### 2.1 Scoping review rationale, objectives, and design

Scoping reviews are useful in investigating exploratory research questions, “mapping” key concepts, identifying and synthesizing evidence, and helping to determine gaps in the existing research (45). Further, scoping reviews can address “broad topics”, as they do not place *a priori* limitations on study type or design (46), and are useful when the subject area and methods of analysis are diverse (47, 48) as is the case with this investigation. Scoping reviews “…require “sense-making” across fields of enquiry that are complex, and which lend themselves to interpretations through many academic and theoretical disciplines” (49) and can help clarify the way forward (50).

The objectives of this scoping review were to:

• identify English and French language peer-reviewed publications reporting empirical findings from studies investigating occupationally injured adult migrants’ experiences and/or outcomes, both internationally and in NZ, from 2007–2022;
• describe key experiences and/or factors associated with occupationally injured migrant women's experiences and outcomes;
• understand the characteristics of migrant women in identified studies;
• identify and describe models, frameworks and theories underpinning identified studies; and,
• identify knowledge (and gaps) related to migrant women injured in the workplace and highlight useful avenues for further research

To address these objectives, the Arksey and O'Malley (46) framework for conducting scoping studies (elaborated by Levac et al. (51) & Colquhoun et al. (45)) was used including: (1) defining the research question(s), (2) identifying relevant empirical publications, (3) selection of publications, (4) charting the data and, (5) collating, summarizing, and reporting findings.

### 2.2 Key concepts and definitions

Levac et al. (51) highlight the importance of clearly defining key concepts, including the target population, concept, and context, in scoping reviews. For this scoping review, the target concept (and context) was “occupational injury”, and “migrants” were the target population. As stated, this paper is focused on reporting findings identified for migrant women.

The definition of occupational injury was based on that of the NZ Accident Compensation Act 2001 (52), including injuries by a worker at their place of employment, or at a location through which the employee moves, and included injuries occurring during work breaks. The terms “occupational injury” and “WRI” were considered synonymous.

A concise definition of the word “experience” is elusive. However, in alignment with the exploratory nature of the scoping review research questions, WRI “experiences” and “outcomes” were broadly defined as instances, events and/or processes surrounding the trajectory of a WRI including (but not limited to) precursors to WRI, working conditions at the time of WRI, the WRI events themselves and events following WRI, including consequences of WRI, interactions, relationships, reflections, and narratives associated with these.

Defining the term “migrant” was challenging, as presently, there is no formal/legal, nor universal definition of the term internationally (53–55). Interpretations are shaped “by geographic, legal, political, methodological, temporal and other factors” (54), have numerous articulations (both formally and informally) in public and academic discourse (53) and can vary considerably between countries (56). Different migrant typologies reflect different aspects of the migration experience including “migrant status, geography, temporality, socio-demographic status and motivations/causal classifications” (57) and can be shaped by geopolitical, legal and methodological imperatives (54).

Definitional inconsistencies with the term “migrant” are compounded with the inclusion of “work” to the typologies. Douglas et al. compiled a list (adapted from the International Organization for Migration (IOM) Glossary on Migration and Organization for Economic Cooperation and Development (OECD) Glossary of Statistical Terms), of terms used to describe migrant workers such as “contract migrant workers, economic migrants, foreign migrant workers, itinerant workers, seasonal migrant workers”, among others (54). Importantly, these terms may exclude occupationally injured migrants for whom the purpose of their initial migration was not work.

The United Nations (UN) defines an international migrant as “…someone who changes his or her country of usual residence, irrespective of the reason for migration or legal status” (58) whereas in NZ, the term “migrant arrival” is used to describe “…an overseas resident who arrives in New Zealand and cumulatively spends 12 out of the next 16 months in New Zealand” (59). For this scoping review, a “migrant” was defined as someone who was clearly identified as born in a country other than the identified host country. This definition was appropriate as, firstly, it aligns with the definition of migrant used in the POIS/POIS-10 studies (9, 60) (within which this scoping review is situated). Secondly, use of this definition mitigates against conflation with findings investigating WRI outcomes and experiences on other mechanisms of disparity, as highlighted previously, including ethnicity, race, citizenship/residency status, and proficiency in the host country official language(s). Whilst acknowledging the importance of these, this investigation is focused on how WRIs are experienced within the host country by foreign-born workers, along with the identification of potential disparities in WRI experiences and outcomes specifically related to being born *outside* the host country and to women migrant workers.

### 2.3 Defining the research questions

The scoping review research questions were developed through consideration of concept (injury), population (migrant women), context (occupational setting) and outcomes (empirically investigated experiences and outcomes) to address the following questions:

• What occupational injury experiences and/or outcomes of migrant women have been reported empirically in peer-reviewed literature, internationally and in NZ?
• What theoretical models or frameworks have underpinned the identified empirical research?
• What are the characteristics of occupationally injured migrant women (e.g., occupational groups, age) and their WRIs (e.g., injury types) in identified studies?
• What key findings have been identified for occupationally injured migrant women including facilitators of, and barriers to, positive outcomes?
• What (if any) knowledge gaps about occupationally injured migrant women's experiences and outcomes are evident from identified studies?

### 2.4 Identifying relevant publications

A two-step process was used to identify peer-reviewed publications reporting findings from empirical research, including quantitative, qualitative, and mixed methods studies on migrants' WRI experiences and outcomes. Firstly, five academic databases were searched following consultation with two subject librarians (from public health and commerce and humanities). Databases included *PubMed* (biomedical, health sciences and related focus) (61), *Cumulative Index to Nursing and Allied Health Literature (CINAHL)* (nursing, allied health, biomedicine and health care) (62), *SCOPUS* (science, technology, medicine and social sciences) (63), *ProQuest Central* (business, health and medical, social sciences, science, and technology) (64) and *Emerald* (Accounting and Finance, Economics, HR and Organizational Behavior, Management Science and Operations) (65). This range of databases was selected in alignment with our scoping review objective of investigating WRI “experiences” (as defined for this scoping review). As such, the aim was to identify studies conducted in a range of settings, including workplace, community, and health, to gain insight into the trajectory of WRI experiences of migrants, including precursors to WRI, WRI events themselves and subsequent experiences. Following searches in the five databases, reference lists of eligible publications were manually searched for further additional eligible publications.

Search terms were developed iteratively, in consultation with the wider research team and subject librarians, through several processes. Firstly, in reference to dimensions of migration, as outlined by Hannigan et al. (55) searches included: “country of origin, length of stay, legal status, citizenship, residency, reason for migration, first language and parental country of birth”. Secondly, glossaries of key international and NZ organizations including the UN (58), the IOM (66), the International Labor Organization (ILO) (67) and Stats NZ Tatauranga Aotearoa (59) were consulted. Thirdly, following test searches in two selected databases, the “subject” filter (in ProQuest) and “subject: major heading” filter (in CINAHL) were applied to ensure further key terms had not been overlooked.

The search included peer-reviewed papers published between January 1, 2007 [aligning with the commencement of POIS (9)] and June, 2022 (the date of the scoping review search). Appendix 1 provides an example of the developed search query and its application in the SCOPUS database.

Identified publications from the five databases were downloaded into *EndNote,* version 20 (68). Duplicates were removed using the *EndNote “*find duplicates” function followed by manual de-duplication. Publication title and abstract screening then commenced, using a developed set of eligibility criteria and a rigorous review process.

The eligibility criteria were developed using the population, context and concept framework and were established both *a priori* (in alignment with the scoping review aims and objectives) and *post hoc*, following more in-depth engagement with identified publications, alongside ongoing consultation with a counter reviewer (the third author, B.C) as described below, and the wider research advisory team, which included the second, fourth and fifth authors (E.W, C.E and S.D). Table 1 presents the eligibility criteria.

**Table 1:** Developed eligibility criteria.

Criteria	Detail	*A Priori* Exclusion	*Post Hoc* Exclusion
Language	Language of publication	Language of publication other than English or French	n/a
Publication type	Type of findings presented	Non-empirical:	n/a
		-reviews of any type *(scoping, systematic, literature, legal/policy, historical, overviews of the field)*	
		*-*conceptual publications, opinion pieces *(letters-to-the editor, commentary, editorial)*	
		-methodology publications	
Population	Age	Participants *exclusively* under 18 years	Includes data for participants *under 18 years of age* (data cannot be disaggregated for over 18 years of age)
Population	Migrant status	Cannot be determined participants were migrants	Includes data for *non-migrants* (data cannot be disaggregated for migrants & non-migrants)
Population	Migrant type	Findings for *non-international* migration	Study population not identifiable as migrating across *international* borders
Context	Occupational	Non-occupational context, injury unrelated to working environment or conditions	Includes data for injuries in *non-occupational* contexts (data cannot be disaggregated for occupational & non-occupational contexts)
Context	National	n/a	Non-OECD country or national context unclear
Concept	Fatalities	Findings confined to fatalities	n/a
Concept	Research focus	Primary focus is not migrants’ outcomes/experiences of WRI or migrants’ working conditions/environment (as contributing to WRI)	Exclusive focus on WRI *incidence,* including comparison of WRI rates of *native- and foreign-born workers*

Prior to title and abstract screening of identified publications, a pilot test was undertaken wherein K.R and B.C independently assessed 100 publications, with regular meetings to ensure the *a priori* eligibility criteria were applied consistently. The small number of discrepancies between reviewers were forwarded to a third reviewer (S.D) for input. Identified French language publications were assessed for eligibility by K.R only, due to language proficiency constraints.

Once title/abstract screening was completed, a set of *post hoc* eligibility criteria was developed to ensure selection of articles at full-text stage closely aligned with the stated aims and objectives of the scoping review. The *post hoc* eligibility criteria were then refined iteratively during several meetings between K.R and B.C, with input from S.D.

Both sets of eligibility criteria (*a priori* and *post hoc*) were then applied to full text screening of publications identified as eligible following title/abstract screening by K.R and B.C, with a small number of discrepancies forwarded to S.D for input.

Following full-text screening, reference lists of eligible publications were manually screened (title/abstracts) by K.R to locate any further relevant publications which met both *a priori* and *post hoc* eligibility criteria as outlined. Eligible publications from both full-text screening and manual screening stages were then rescreened by K.R to identify studies with substantive findings specifically for migrant women, including findings beyond incidence/prevalence of WRI alone.

### 2.5 Charting of findings

Key characteristics of identified eligible publications were “charted” (46) by K.R in alignment with the scoping review objectives. Firstly, the full bibliographic reference and full text of each eligible publication was entered into *NVivo* (v. 1.6.1) (69) data management software, to help record descriptive characteristics of publications, in alignment with the developed scoping review research questions. Table 2 presents these characteristics extracted by category and aspect. The data managed in *NVivo* (v. 1.6.1) (69) was then used to chart detailed notes and reflections for each publication. Findings were then synthesized narratively, including key findings, limitations, and ways forward.

**Table 2:** Descriptive characteristics of identified eligible publications (*n* = 22^a^) by year published, study methods, country, proportion of women participants, occupation, participant COB and theoretical frameworks reported.

	Identified publication(s)	Total publications
Year published		
2007–2010	(33, 70)	2
2011–2015	(20, 21, 40, 71–74)	7
2016–2020	(4, 18, 23, 75–79)	8
2021–2022	(22, 80–83)	5
Study methods		
Qualitative	(4, 18, 20–23, 70, 72, 73, 75–78, 81–83)	16
Quantitative	(33, 40, 71, 79)	4
Mixed	(74, 80)	2
Country		
Australia	(74, 79)	2
Canada	(18, 40, 72, 73, 76, 77, 83)	7
Spain	(23, 82)	2
USA	(4, 20–22, 33, 70, 71, 75, 78, 80, 81)	11
Proportion of women participants		
100%	(18, 20, 23, 71, 75)	5
50–99%	(4, 21, 33, 70, 74, 79, 81)	7
≤50%	(37, 40, 72, 73, 76–78, 83)	8
Unstated	(80)	1
Occupation		
Commercial cleaning, hotel housekeeping	(4, 33, 75)	3
Dairy farming	(22, 78, 80)	3
Homecare, direct care, domestic workers (paid)	(18, 23, 81, 82)	4
Hospitality, service workers	(70, 82)	2
Other, manual	(21, 71)	2
Poultry processing	(71)	1
Various occupations	(20, 21, 40, 72–74, 76, 77, 79, 83)	10
Participant COB		
COB unstated	(18, 33, 40, 73, 74, 77, 80)	7
China, Taiwan, Hong Kong	(70)	1
Mexico	(20–22, 75, 78)	5
Philippines	(18)	1
Latin America (COB unspecified)	(20, 71)	2
Various COB	(76, 79, 82, 83)	4
Various COB (Africa)	(81)	1
Various COB (Central, South America)	(4, 20–23, 72, 75, 78)	8
Theoretical frameworks reported		
Karasek's “job demand-control/support” model (84, 85) & extensions	(71, 75)	2
Worker OSH rights & Marshall's “social citizenship” (86)	(72)	1
Goffman's theory of “social stigma” (87)	(76)	1
Psychosocial stress approach (88)	(21)	1
Frameworks of precarious work & health inequity (89, 90)	(80)	1
De Genova's “deportability in everyday life” (91)	(22)	1
Mullins, “intersectionality theory” (92)	(81)	1
Symbolic rupture, Edelman (93), extended by Denizeau (94)	(77)	1

^a^ From 21 studies (77); and (76) reported findings from the same study.

## 3 Results

### 3.1 Identified eligible publications

**Figure 1:**
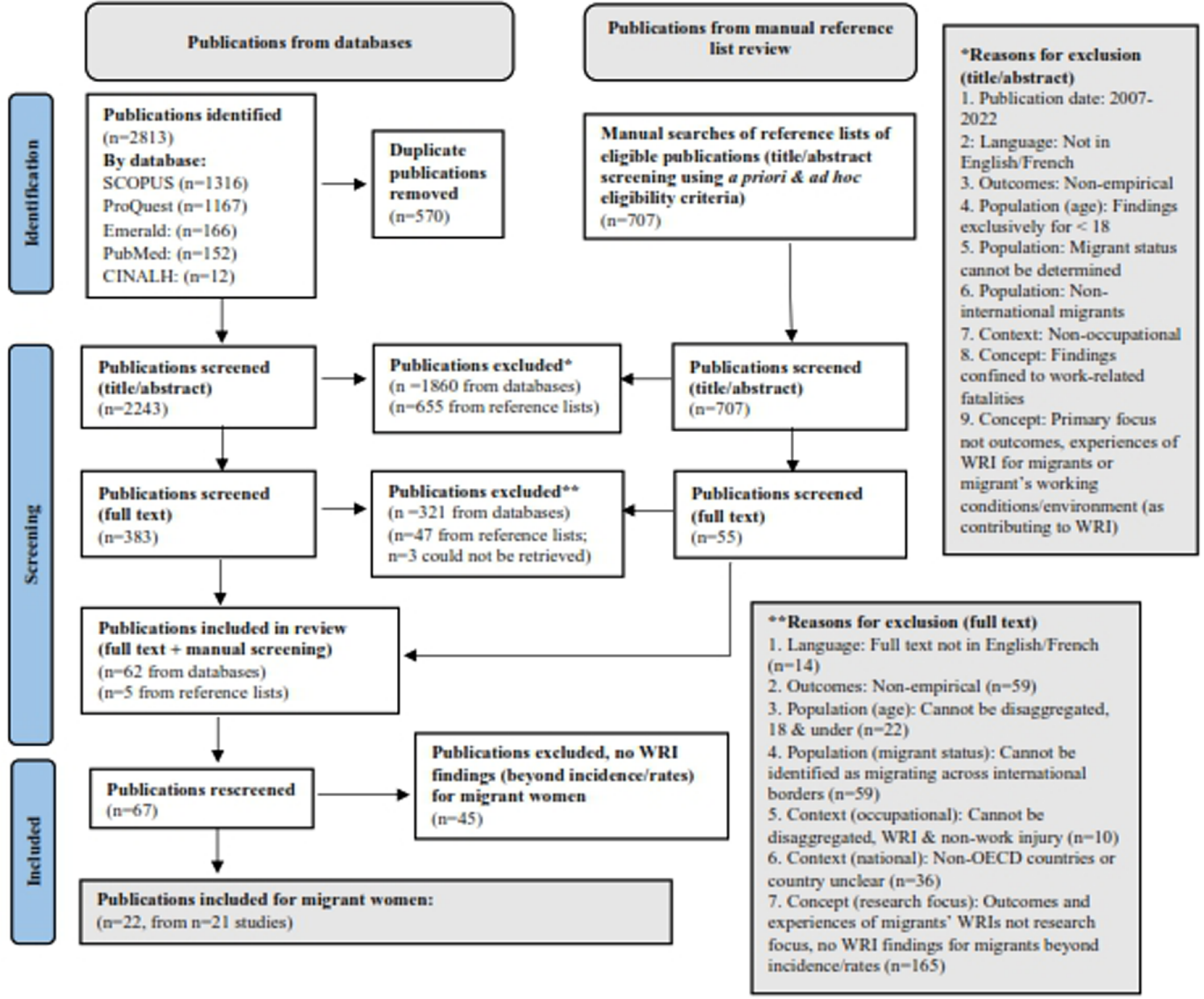
PRISMA diagram, identification of publications from databases and other sources (95).

A total of 2,813 publications were identified for review from the five selected databases. A PRISMA diagram (95) is presented in Figure 1, illustrating the identification, screening, exclusion criteria and numbers of papers.

From the 2,813 publications, 570 duplicate publications were removed, with 2,243 remaining for title/abstract screening. Following title/abstract screening, 1,860 publications were identified as ineligible for full-text screening. Full text screening of the remaining 383 publications retained 62 for final inclusion.

The manual search of reference lists led to the inclusion of a further 5 eligible publications, bringing the final total to 67 included publications. These were rescreened to identify publications reporting findings specifically for migrant women. Through application of this criteria, 45 further publications were excluded, leaving 22 publications (based on 21 studies) with findings for WRI experiences and outcomes for migrant women.

### 3.2 Descriptive characteristics of eligible studies

The 22 publications, from 21 distinct studies, were classified by year published, study type, country, occupation, participant country of birth (COB) and theoretical framework (if reported) (Table 2). Some publications included participants from more than one COB and/or occupation, hence the total does not always equal 22.

Identified publications with findings for occupationally injured migrant women came from four OECD countries: the United States (US) (*n* = 11), Canada (*n* = 7), Australia (*n* = 2), and Spain (*n* = 2); none were identified from NZ. A range of study designs were identified, including qualitative (the majority), quantitative and mixed-methods studies. Of the 21 distinct studies including findings specifically for migrant women, only five studies focused *exclusively* on the experiences and outcomes for women (18, 20, 23, 71, 75). Table 2 presents the proportion of women participants in identified studies.

### 3.3 Theoretical models/frameworks

Regarding explicit consideration of theoretical models underpinning research, nine of the 22 publications explicitly referred to theory or theories. Arcury et al. (71) applied Karasek's job-demand-control-support model (84, 85, 96) (with extensions) to assess perceived support from supervisors (97). This included using items from the “social power scale” (98) and work safety climate from the “perceived safety climate scale” (99) to investigate relationships between work organization and musculoskeletal injuries among Latina immigrant manual and poultry processing workers in the US. Aspects of work organization were associated with epicondylitis (awkward posture and decision latitude), rotator cuff syndrome (awkward posture and psychological demand), and carpal tunnel syndrome (job strain, psychological demand, decreased skill variety and job control), however, the cross-sectional study design, did not allow definitive identification of these injuries as work-related (71). Johnson et al. (100) extended Karesek's model (101, 102) by including the influence of social support at work. In their US study with Latina hotel housekeepers, Hsieh et al. (75) applied this extended framework, including “social hazards” as contributing to work-related health and wellbeing outcomes for these workers.

Panikkar et al. (80) analyzed the occupational health hazards and outcomes of migrant dairy farm workers in Vermont, US, informed by frameworks of precarious work (89, 90) as contributing to health disparities (including occupational hazards) for this group. “Social hazards” were both the product of and contributors to precarity, including immigration stress, language constraints, and access to safe housing, transport and healthcare (80). These contributed to physical and social isolation and unfavorable “power dynamics” with employers, constraining health management choices, including following WRI (80).

Côté et al. (76) applied Goffman's frameworks of stigmatization, identity and social interaction (87) to investigate stigmatization experienced in the return-to-work (RTW) trajectory among occupationally injured migrant workers in Montreal, Canada, including how these are operationalized across a range of interactions, relationships and contexts (103). Using their developed conceptual model of “cumulative stigma” (76), they found overlapping and interconnected forms of stigma to be both products of, and contributors to, negative experiences in the RTW trajectory. They highlight that cumulative stigma may contribute to a “cumulative labelling effect” (76) by health care professionals and RTW advisors, thus potentially contributing to WRI treatment inequities for migrants, including migrant women.

Marshall's (86) conceptualization of “citizenship” encompasses civil, political and social rights. Basok et al. (72) use this framework to consider occupational safety and health (OSH) as a fundamental “social right”, under which the right to a safe work environment is based on “collective equality” rather than individual freedoms. Their study identified a gap between having OSH rights and the actual assertion of these in WRI reporting, along with highlighting access barriers specifically for migrant workers in Ontario, Canada.

A “psychosocial stress approach” (88) was used by Martinez et al. (21) to investigate occupational stressors associated with racism and discrimination and how these influence the risk of WRIs for immigrant day laborers in Baltimore, US. Identified stressors included; stress over meeting deadlines, potential wage theft, sudden dismissal, and workplace immigration enforcement (21), with the adverse influence of these on mental health highlighted, along with risk of physical WRI (21).

Covington-Ward investigates the “physical embodied costs” (81) experienced by African migrant care workers in Pittsburgh, US, including WRIs, through the framework of “intersectionality” and the “Sojourner Syndrome”, which consider the intertwining of race, gender and class as contributing to health disparities (92). The physical toll of the work (WRIs, pain, physical exhaustion, low energy levels) contributes to a lack of social mobility for migrants, by hindering motivation and capacity for upskilling and subsequent transition to less risky employment (81). As such, migrants have undertaken migration journeys, only to find themselves rendered immobile in terms of social mobility (81). The extent to which these mobility constraints are simultaneously precursors to, and the product of the WRI trajectory for migrant women, requires further investigation.

Sexsmith (22) addresses how precarity shapes OSH for undocumented migrant dairy farm workers in New York, US, through the framework of “deportability in everyday life” (91). Through this, the *potential* for deportation, along with industry imperatives, production processes and low regulation, were found to contribute to physical work-related risks including WRIs and chronic pain (22). The extent to which being or becoming “documented” (having or obtaining legal status in the host country) mitigates or attenuates the risks associated with “deportability” for migrant women workers, remains unclear.

Gravel et al. (77) investigate the motivation of migrant workers in the RTW process following WRI, based on a model of precarity (104), including job precarity (contract type), immigration-related precarity, professional precarity (non-recognition of previous work qualifications and experience) and economic precarity (unpaid overtime, low hourly wages regardless of qualifications, debts owed to recruiters/agents and overseas remittances). They conclude these dimensions of precarity may be potential determinants of a “symbolic break” with the RTW process, negatively influencing WRI experiences and outcomes (77).

### 3.4 Categorized findings for women from eligible publications

Findings from eligible publications related to migrant women's experiences and outcomes of WRIs were reviewed and grouped into five overarching “topic areas” for presentation including working conditions, legal rights, identities, the role of gatekeepers, and precarity, each of which are summarized below.

#### 3.4.1 Working conditions

Identified studies described working conditions experienced by migrant women and how these contribute to potentially unsafe work environments and in some cases, WRIs. Aspects of work conditions variously included: (i) work background to job mismatch, (ii) work demands including task functions, working hours and work rate, (iii) OSH training and personal protective equipment (PPE) provision, and (iv) aspects of working conditions specific to women workers.

##### 3.4.1.1 Work background to job mismatch

Premji and Smith (40) found male immigrants to Canada who were over-educated for their jobs were over three times as likely to experience a WRI, however, this was not found for over-educated women migrant workers. Nevertheless, in other studies, migrant women workers' pre-migration educational/work qualifications, skills and experience were reportedly underutilized in the host country work environment, including reports of “funneling” (20) of women migrants into manual and service occupations, requiring skills incommensurate with their previous work experience (20, 73, 82) and potentially, increasing their risk of WRI.

##### 3.4.1.2 Work demands

WRIs were associated with the organization of work environments for women migrant workers, including reduced worker autonomy and decreased variety of job function (71) and with excessive workload and work intensification, including overlapping job responsibilities, particularly in paid caregiving (4, 18, 20, 23, 82). In other occupations, including homecare/domestic work, commercial cleaning and farming, the expected work rate and “anxiety to beat the deadline” (21) contributed to stress, and OSH risks (18, 20, 21, 75). Further, excessive work hours were reported by migrant women participants, including 12–16 h shifts and split shifts (22, 23, 82), contributing to work-related fatigue.

##### 3.4.1.3 OSH training

In some studies, OSH training was reportedly not provided to migrant women workers or was insufficient (23, 80, 82) and when provided, at times, consisted of informal instruction by co-workers during work tasks (80). OSH resources did not consider employees linguistic proficiency (74, 75), which contributed to lack of recognition and reporting of accidents (74). PPE equipment was not provided (20, 23) or needed to be purchased “out of pocket” in the case of migrant women hotel housekeepers (75) and there were reports of a lack of workplace ergonomic adaptation for women (23). Shankar et al. (83) report that when OSH training *did* occur, workplace bullying, and harassment were not recognized as WRIs.

##### 3.4.1.4 Aspects of working conditions specific to women

Regarding working conditions, as women, migrant workers reportedly faced increased risk of workplace harassment and sexual harassment, including from supervisors (20, 21, 83) along with discrimination and dismissal due to pregnancy (20). These risks occurred alongside the reported lack of OSH acknowledgement of bullying and harassment as WRIs (83) as outlined. Further, in roles as primary caregivers of children, undocumented migrant women workers faced additional work-related stress surrounding potential immigration enforcement, including inability to arrange childcare if detained (21).

#### 3.4.2 Legal rights

Lack of worker awareness of their OSH legal rights influenced WRI experiences and outcomes, such as knowledge of what is considered a WRI, and processes following injury, including an understanding of workers' own rights but also of employer WRI obligations (74). Some employers acted to mitigate exposure of WRIs including by paying for medical expenses themselves (circumventing WRI reporting and compensation) with migrant women workers interpreting their actions as acts of kindness (73). Importantly, however, *awareness* of workers' OSH rights did not necessarily translate into workers asserting these, as identified for migrant women caregivers in Southern Spain (23) and highly-educated workers in Alberta, Canada (83).

#### 3.4.3 Identities

Migrant identities shaped WRI experiences and outcomes including aspects of work-related risk and working conditions, WRI reporting and seeking treatment, and the RTW process. Self-identifying as “migrants” (vis-à-vis native-born workers) shaped tolerance of work-related risk. Pride in being hardworking (and working harder than native-born co-workers) reportedly contributed to task intensification and unsustainable expectations of work rate (20) for migrant women, alongside self-perceptions as tolerant of workplace risks (72).

Further, overseas remittances sent by migrant women workers to family in their country of origin contributed to the “socially elevating” aspect of migrant identity (23), with this potentially jeopardized for themselves and their families by reporting OSH concerns and/or seeking treatment for WRIs (23).

#### 3.4.4 The role of “gatekeepers”

The influence of key gatekeepers in the WRI experience was highlighted including: (i) employer and supervisor attitudes and the OSH environment (ii) employer practices following WRI, and (iii) attitudes of WRI compensation advisors.

##### 3.4.4.1 Employer and supervisor attitudes and the OSH environment

Discriminatory attitudes from employers, supervisors, and co-workers contributed to migrant women workers' OSH environments in a range of ways (20, 83). Workers who highlighted OSH risks were seen as “troublemakers” and feared reprisals for doing so (72, 83). Some supervisors expressed irritation when needing to repeat OSH information, with workers feeling at fault if they did not understand (83). In paid caregiving work, a clear prioritization of the well-being of care recipients over that of workers was identified (81).

##### 3.4.4.2 Employer practices following WRI

Some employers did not report WRIs or seek treatment for those sustaining these (74). Further, Latina immigrant workers were reportedly denied time off for treatment/recovery from WRIs (20) and, in some cases, employers pressured migrant women workers for early return-to-work (RTW) following injury (73). Reprisals for taking time off for WRIs experienced by migrant women workers included less favorable work rosters and more difficult tasks (75) and in some cases, dismissal (72).

Regarding WRI compensation, whilst some employers paid injured migrant women dairy farmworkers above and beyond workers’ compensation payments for medical treatment and time off, others refused to pay for WRI-related recovery time (78). Some employers of migrant women hotel housekeepers made no effort to ascertain the cause of WRI, indicating a lack of concern for safe work environments and attention to WRI prevention (75).

##### 3.4.4.3 Attitudes of worker's compensation advisors

Analyses of worker compensation advisors’ views towards RTW following WRI, report the intertwining of age and gender identities as influencing older migrant women migrants' motivation to RTW following WRI (76, 77). They suggest migrant women's identities as “self-assigned family caregivers” may take precedence over their identities as workers, negatively impacting motivation to RTW following WRI (76, 77). This conflicted with a report that an occupationally injured migrant woman viewed her WRI as an opportunity to retrain and upskill (to a job more commensurate with her skills/previous experience), however, was not supported by RTW advisors (77) to do so.

Further, worker's compensation advisors reported “self-stigmatization” amongst occupationally injured migrant women, stating that they perceived themselves to be at greater risk of disability due to their WRI than their native-born counterparts (76).

#### 3.4.5 Precarity

For migrant women workers, precarity influenced the overall work experience, from work conditions to WRI reporting, treatment, and health management choices. This included: (i) socio-economic precarity, and (ii) notions of “replaceability”.

##### 3.4.5.1 Socio-economic precarity

Socio-economic precarity was highlighted as a key aspect of migrant women's work trajectory and WRI outcomes and experiences. In a study of migrant women hotel housekeepers, 18 of 21 participants reported an annual income below the US federal poverty line, with many having second jobs (75). Low pay was highlighted (74, 80) including less than minimum wage (74) and specifically along gendered lines: “That's how we experience discrimination in the workplace, low wages. Especially if you’re a woman, you’re going to get the lowest possible amount, even if you’re doing the same or harder work than men, you’re going to get a really low wage” (80).

Regarding working conditions, socio-economic precarity was experienced by migrant women workers through task intensification without commensurate compensation (20, 80), excessive work hours (22, 23, 82), split shifts (22), unpaid overtime (75) and wage theft (21).

Workers continued working through WRIs due to financial precarity, including the need to support family and pay the bills (75, 82), with some not paid for time off following WRI (78). Further, women migrant dairy farm workers reported experiencing housing precarity through needing to leave employer-owned accommodation if unable to work (78).

Migrant women's health management choices shaped by financial precarity included presenteeism following WRI due to fear of job loss (23, 82), avoidance of medical treatment due to having to pay own medical bills and/or lack of health insurance (75), and being unable to take time off due to needing to support children (82). In the absence of medical care, workers relied on self-management for symptoms, including traditional medicines from their country of origin (20, 23) along with other “coping strategies”. (70, 75).

##### 3.4.5.2 “Replaceability”

Migrant women workers experience precarity over fears of employer reprisals and of being “replaceable” as workers. These fears can inhibit workers from raising OSH concerns (23, 72, 74) and shape experiences following a WRI, including presenteeism following injury (23, 82) and WRI under-reporting (23, 72). Perceptions of replaceability emerge from both employers' attitudes *and* workers self-identification as “replaceable” (72, 75) and can contribute to worker's “prioritizing job security over health and safety” (74) due to fears over dismissal or contract non-renewal (72).

## 4 Discussion

### 4.1 Synthesis of findings, including research gaps identified for women migrants

As mentioned, identified studies came from four OECD countries only, with none from NZ. This is concerning due to the comparatively high percentage of the NZ population reported to be foreign-born (28.7%) (1), of which 51.6% were women (105). Of the four countries for which findings were identified, only Australia's foreign-born population exceeded this figure at 30.1%, with Canada, the US and Spain substantially lower at 21.3%, 15.3% and 14.6% respectively (1). Further, differences in immigration settings, WRI compensation systems [including NZ's ACC system (106)] and health care systems (universal/publicly funded vs. user-pays/private), potentially inhibit direct comparisons between NZ migrant women's WRI experiences and those from other identified countries, creating impetus for NZ research.

Despite the range of study designs in identified publications, only three included findings for longer-term experiences and outcomes of WRIs (76, 79, 80), whilst three studies called for such longer-term data (71, 75, 78). Further, although a range of theoretical frameworks were applied, there was a paucity of analyses of the WRI experience through life-course/trajectory models, highlighting a gap in understanding of how these experiences are “lived” over the long term by occupationally injured migrant women.

### 4.2 A matryoshka story: a framing narrative of women migrants' WRIs

A framing narrative, or, a “story within a story” offers context and clues to interpretation of the “main narrative” including why the “story” needs to be told (107). The metaphor of a “matryoshka doll” (nested dolls) was chosen by us to interpret the framing narrative of migrant women's WRIs as, based on the findings of this scoping review, migrant women's WRI experiences can be conceptualized as “nested within” (or connected to) their wider work and life contexts, or as “stories” located within the overarching framing narrative. This provisional analytical framework was selected based on consideration of three concepts: uniformity, femininity, and appropriation.

Firstly, regarding uniformity, Matryoshka dolls, although seemingly uniform, were, historically, the product of individual craftsmanship, with each set rendered differently (108); as such, this metaphor rejects the essentialization of migrant women's WRI experiences. Regarding femininity and appropriation, the “hyperbolically feminized” (109) matryoshka symbolizes female fertility, however, such representations potentially originate from the artisans who construct them. Furthermore, although often perceived to have originated in Russia, matryoshka, were in fact, developed from a Japanese nested toy (108, 110). This metaphor is therefore appropriate to illuminate occupationally injured migrant women's potentially “hidden” stories, challenge assumptions, and reject appropriation of these experiences (i.e., by having these stories told by migrant women themselves).

Models using a matryoshka metaphor have been applied in other contexts, including to interpret geopolitical relationships (111) and penal decision-making (112), amongst others. To the best of our knowledge, this conceptual framework has not yet been applied to interpret WRI experiences. The provisional (to be refined following forthcoming qualitative interviews with occupationally injured NZ migrant women) matryoshka analytical model was developed based on the key topic categories synthesized from findings from identified publications in this review including: (i) broader working conditions experienced by migrant women, (ii) legal rights (including those related to safe work), (iii) the role of intertwining identities as both migrants and women, (iv) the influence of key gatekeepers throughout the WRI trajectory, and (v) the impact of precarity on WRI experiences and outcomes. These topic categories are summarized below, along with suggested “ways forward” to address identified gaps in understanding migrant women's WRI outcomes and experiences.

### 4.3 Working conditions

Firstly, a “funneling” of migrant women into “risky occupations” (20) was identified, with lack of recognition of previous qualifications, education and job experience from their country of origin. For *male* migrants, being over-educated for a job reportedly doubles the risk of WRI, with suggested reasons for this including; tendency of over-educated workers to underestimate risks, lower job satisfaction (leading to greater staff turnover) and less solidarity with co-workers (40). An association between over-education to job-mismatch and risk of WRI was not identified for *women* migrants (40). However, the extent to which over-education impacts experiences and outcomes *following* WRI and specifically for women migrants, requires further investigation as being over-qualified for a job could negatively impact mental health and motivation, including potentially, RTW motivation following WRI.

Further, although alluded to by Gravel et al. (77), RTW trajectories following WRI have not been investigated through an “opportunities” lens, or, as potential moments of intervention to “reskill” migrant women workers into jobs more commensurate with their previous work experience or to the needs of the job market. Investigation into the views of RTW and WRI compensation experts and importantly, occupationally injured migrant women themselves, is needed to determine both the feasibility and desirability of reimagining the RTW process in this way.

Regarding working conditions, the acknowledgment of workplace bullying as a recognized OSH risk [rather than an employment issue, see Millis (113)] may be significant for women migrant workers, due to the impact of this reported in international studies (20, 83). In NZ, the Health and Safety At Work Act (2015), by mandating workers “be given the highest level of protection from workplace health and safety risks…” (114) offers provision for addressing workplace bullying (113). Alongside this, and despite the acknowledgement of workplace bullying as a “serious and common” health risk for NZ workers (115), and particularly for women workers (116), a “laissez-faire” (117) approach towards this has been reported in NZ. Given this, further research should include an assessment of the extent to which workplace bullying has shaped the WRI experiences of migrant women, both in NZ and overseas. Further, the extent to which these are formally recognized and “operationalized” as OSH risks in legislation in other OECD countries warrants investigation, potentially through a scoping review. Lastly, participatory action approaches (118) should be used *with* women migrant workers themselves (both in NZ and overseas) to ascertain awareness of workplace bullying as an OSH risk and the extent to which this is incorporated into OSH training for this group (across diverse industries), with the aim of identifying current gaps (if any) in training and investigating how potential shortcomings should be remediated.

### 4.4 Legal rights

Basok et al. (72) report knowledge of OSH rights does not necessarily translate into assertion of these by migrant workers, in Ontario, Canada. Investigation into the context-specificity of their findings, and specifically, their applicability for migrant women workers in NZ, is warranted. This could include assessment of migrant women workers knowledge of OSH rights and employer obligations using case study methodology to explore outcomes across a range of industries, followed by an assessment of barriers and facilitators to the access and exercise of these OSH rights for migrant women, if/as identified.

### 4.5 Identities

This review has highlighted the role of identities in WRI experiences for migrant women workers, including both gender and migrant identities, and the intertwining of these. Previous research has explored the role of gendered identities such as “machismo” in workplace exploitation (119) and WRIs for migrant *men* (120), however, further investigation is needed to determine the extent to which gender identities shape *women* migrants' WRI outcomes and experiences.

Further, RTW advisors reportedly view women migrants' (particularly older women) RTW motivation following WRI as negatively influenced by gendered identities as family caregivers (rather than identifying primarily as workers) (76, 77). Additionally, RTW advisors noted occupationally injured women migrants saw themselves “…as being more disabled” due to their WRI than their native-born peers (76). As (to the best of our knowledge) these perceptions have not been substantiated with occupationally injured migrant women themselves, it is unclear whether this reflects self-stigmatization (as implied by the RTW advisors) or, stigmatization of these workers by the RTW advisors *themselves.* Further research should explore this, as advocated by Côté et al. (76).

Further, the WRI experiences of migrant women were reportedly shaped by identities as “migrants”, including those grounded in “model minority discourse” (121, 122) or attributes of a “good migrant” identity (123) including stoicism, a strong work ethic, and as working harder and faster than their native-born counterparts (20). Further investigation is required to determine the interplay between these migrant identities and WRI experiences, given work intensification has been identified as “an occupational hazard” for migrant women (18).

Côté et al. (76) report “cumulative stigma” for migrants following WRI, based on ethnicity, culture, language, socio-economic status, gender, along with stigma related to having a WRI itself. This includes self-stigmatization due to shame resulting from the WRI and a lack of understanding (76). Gravel et al. (77) call this trajectory “un parcours déshonorant” (a journey of shame). Alongside this, the migrant identity includes perceptions of the migration experience as “socially elevating” for migrant women (23). Further investigation is therefore needed to ascertain how this potential dual stigmatization (due to the WRI itself *and* the potentially negative impact on the wider immigration trajectory) influences WRI experiences and outcomes of occupationally injured migrant women.

### 4.6 The role of “gatekeepers”

Gendered occupations such as caregiving (124, 125), paid domestic work (126, 127) and home cleaning can be characterized as “invisible” in that they take place away from the public eye, and often during unusual hours (128). Invisibility may also be significant for migrant dairy farm workers, some of whom are socially and physically isolated on dairy farms, many without transportation or access to community resources (80). Migrant women in these “invisible” occupations may experience “double isolation” (124) through both their status as migrants and as workers that are “invisible and undervalued” (124).

Social isolation (along with precarity) may contribute to over-reliance on “gatekeepers” such as supervisors and employers during WRI treatment (80) and reporting (73). Other “gatekeepers” in the WRI trajectory, potentially include WRI compensation and RTW advisors (76, 77) along with health care professionals (129). Little appears to be known about the extent (or potential of) such “gatekeepers” to act in a protective role for migrant workers, either in a prevention capacity or as advocates following WRI. In NZ, interactions with “front-line” health providers during presentations for health issues (including WRIs) have been proposed as potential key moments of intervention in workplace exploitation and modern slavery (130). The feasibility and acceptability of such advocatory interventions to both providers (and including, in NZ, ACC) and migrant service users requires further investigation.

### 4.7 Precarity

Precarious socio-economic status reportedly influences the WRI experience for migrant women in a range of ways including treatment avoidance (75, 82) and subsequent reliance on self-management of WRIs (70, 75). It is unclear whether WRI treatment avoidance due to socio-economic concerns occurs in other countries, including those with publicly funded health care and specifically, in NZ, given the ACC no-fault injury insurance compensation scheme (106). Even in NZ, however, a general practitioner may be the first point of contact with health care following a WRI, for which service users must pay consultation fees (with some users receiving subsidies) (131). Given the noted impact of socio-economic status on the WRI experience, the extent to which such fees create barriers to initial treatment-seeking for occupationally injured migrant women workers requires further investigation.

### 4.8 Strengths and limitations

This scoping review, using cross-disciplinary databases and an inclusive search strategy, facilitated identification of a broad range of publications and subsequently, synthesis of findings from diverse study designs and methods, contributing to useful findings. Nevertheless, as with all reviews, there were some limitations.

Firstly, publications identified at both title/abstract and full-text stages in languages other than English or French could not be reviewed due to linguistic proficiency constraints of the reviewers. Further, although identified French language publications were reviewed, exclusive use of English language databases potentially hindered identification of other important French language publications. In addition, the review timeframe of 2007–2022 may have excluded important earlier and very recent findings.

Importantly, as noted in Table 2, 15 of 22 identified eligible publications reported findings for *both* men and women migrants. At times, the disaggregation of findings specifically for women within some publications, was challenging. A noted limitation of scoping reviews is that findings can only be analyzed as reported, therefore, potentially important information related to findings for women may have been overlooked, despite the systematic approach taken to the extraction of findings for women.

Finally, a review of grey literature was not feasible due to time constraints (given the scoping review is one component of the larger PhD study) alongside extensive variation in WRI organizations, WRI compensation systems and indeed, health-care systems in general across the OECD. Such a review of existing grey literature on migrant women's WRIs would be a useful future investigation, both for NZ, and internationally for OECD countries.

## 5 Conclusion

This scoping review of empirical publications reporting findings on the WRI experiences and outcomes of women migrants from OECD countries, has highlighted a relative paucity of studies on this topic over the target timeframe. Further, no NZ studies were identified as eligible at the full-text screening stage.

As described, as part of the wider project within which this scoping review is nested, a review of NZ media representations of migrants WRIs was undertaken (12) identifying a “default conceptualization” of WRIs as experienced mainly by migrant *men* (rather than their women migrant counterparts). These findings have largely been replicated in this scoping review of empirical studies, with few identified studies being found to focus specifically on the experiences and outcomes of women migrants.

Findings from this scoping review were synthesized and reported through a provisional analytical model, the “matryoshka framing narrative”, which is being developed to interpret and understand the wider lived experiences of migrant women who experience WRIs, and which will be refined through forthcoming qualitative interviews with occupationally injured migrant women in NZ, alongside investigation into some of the key knowledge gaps highlighted in this scoping review. In addition, we recommend this as an important avenue of research for other countries, given the relative lack of empirical findings identified for migrant women from other OECD countries in this scoping review. Although scoping reviews can be considered an important step in identifying key knowledge gaps in the literature (46), in this case, further investigation is required to ascertain how the experiences of occupationally injured migrant women are “lived” in different countries and contexts.

The matryoshka model (although preliminary) reflects the complicity of a multitude of influences in the WRI experience, potentially specific to this group, suggesting the consequences of WRIs may be uneven, with migrant women experiencing different, and potentially, greater disparities in outcomes. These findings create impetus to investigate knowledge gaps highlighted in this review, with a view to urgently addressing potential disparities in WRI outcomes for migrant women specifically, as identified.

## Data Availability

The raw data supporting the conclusions of this article will be made available by the authors, without undue reservation.

## Appendix 1: Boolean search query and sample search (SCOPUS)

**Table:**

Developed Boolean Search Query
*"migrant worker*” OR “immigrant worker*” OR “foreign worker*” OR “seasonal worker*” OR “undocumented worker*” OR “refugee worker*” OR “non-citizen worker*” AND “occupational injur*” OR “occupational accident*” OR “work-related injur*” OR “workplace injur*”*
Application of Search Query in SCOPUS Database
"migrant worker*” OR “immigrant worker*” OR “foreign worker*” OR “seasonal worker*” OR “undocumented worker*” OR “refugee worker*” OR “non-citizen worker*” AND “occupational injur*” OR “occupational accident*” OR “work-related injur*” OR “workplace injur*” AND (LIMIT-TO (PUBYEAR, 2022) OR LIMIT-TO (PUBYEAR, 2021) OR LIMIT-TO (PUBYEAR, 2020) OR LIMIT-TO (PUBYEAR, 2019) OR LIMIT-TO (PUBYEAR, 2018) OR LIMIT-TO (PUBYEAR, 2017) OR LIMIT-TO (PUBYEAR, 2016) OR LIMIT-TO (PUBYEAR, 2015) OR LIMIT-TO (PUBYEAR, 2014) OR LIMIT-TO (PUBYEAR, 2013) OR LIMIT-TO (PUBYEAR, 2012) OR LIMIT-TO (PUBYEAR, 2011) OR LIMIT-TO (PUBYEAR, 2010) OR LIMIT-TO (PUBYEAR, 2009) OR LIMIT-TO (PUBYEAR, 2008) OR LIMIT-TO (PUBYEAR, 2007)) AND (LIMIT-TO (LANGUAGE, “English”) OR LIMIT-TO (LANGUAGE, “French”)) AND (EXCLUDE (DOCTYPE, “ed”) OR EXCLUDE (DOCTYPE, “le”))
